# The feasibility and acceptability of using EMA and physiological data to measure day-to-day occupational stress, musculoskeletal pain and mental health

**DOI:** 10.1186/s13104-024-06950-1

**Published:** 2024-10-02

**Authors:** Victoria Weale, Jasmine Love, Els Clays, Jodi Oakman

**Affiliations:** 1 Centre for Ergonomics and Human Factors, Department of Public Health, La Trobe University, Bundoora, VIC 3083 Australia; 2 Judith Lumley Centre, School of Nursing and Midwifery, La Trobe University, Bundoora, VIC 3083 Australia; 3 Department of Public Health and Primary Care, Ghent University, C. Heymanslaan 10, Ghent, 9000 Belgium

**Keywords:** Ecological momentary assessment (EMA), Physiological data, Feasibility, Acceptability, Protocol, Occupational stress

## Abstract

**Objectives:**

This study aimed to assess the feasibility and acceptability of using EMA questionnaires and physiological data via wristbands to measure day-to-day occupational stress, musculoskeletal pain, and mental health among university employees (*N* = 23), across 10 work days. Adherence to the study protocol as well as participant experiences (via semi-structured interviews) with the protocol were used to assess feasibility and acceptability of the method.

**Results:**

Adherence to the study protocol was excellent. Participants wore the wristband for a mean of 9.7 days. Participants completed a mean of 24.5 EMAs (out of 30). Semi-structured interviews with participants revealed that a small number of participants had difficulties uploading data from the wristband. The timing of EMAs was challenging for some participants, resulting in missed EMAs, raising questions about whether EMA frequency and timing could be changed to improve adherence. Some EMA items were difficult to answer due to the nature of participants’ roles and the work undertaken. Overall, the protocol was feasible and acceptable but highlighted future potential changes including using a different physiological data collection tool, reducing the number of EMAs, adjusting EMA timings, and reviewing EMA items.

## Introduction

Ecological Momentary Assessment (EMA) involves repeated data collection to capture participant behaviour and experiences in real time in the natural environment [1]. EMA can overcome limitations of retrospective reports, such as recall issues, and allow for variation and changes in behaviour and experiences at different times to be assessed [2].

Continuous physiological monitoring (CPM) of key physiological indicators such as heart rate and electrodermal activity (e.g., sweating) can be done using non-invasive methods such as wristbands. For example the Empatica^®^ E4 wristband measures skin temperature, heart rate, blood volume pulse, electrodermal activity, and accelerometry and has been used to explore a range of health issues, including stress and seizure detection [3–6], atrial fibrillation episodes [7], and to predict aggressive behaviour [8].

A previous study used both EMA and CPM to investigate exposure to day-to-day work environment risk factors, stress outcomes, and stress and health related behaviours [9, 10] of European workers in academia. The study protocol required participants to complete EMA questionnaires and wear an Empatica^®^ E4 wristband for waking hours across 15 working days, with EMA questionnaires delivered via an Android smartphone app at approximately 90 min intervals across the work day, with an additional EMA questionnaire in the evening [4]. Participant adherence to protocol was reported to be good and participants did not find the protocol burdensome; however, participants did report that it was sometimes difficult to complete the EMAs (e.g., due to work commitments) and some items were confusing or difficult to answer if they appeared irrelevant (e.g., items about stressful situations that had not occurred in the day) [11].

An unpublished study by Talbot [12] tested the feasibility and acceptability of the study protocol developed by Bolliger et al. [4] in an Australian context with employees in a private engineering firm. Feasibility is defined as whether something can and should be accomplished, and if so, how [13], whereas acceptability concerns protocol reception by the study population and how well it meets the needs of both the study population and the research team [14]. Talbot reported that whilst some elements of the protocol (baseline questionnaire and wristband use) were feasible for study participants, the feasibility and acceptability of the EMAs were low due to the high frequency of surveys (up to eight per day), survey items being unclear, and incorrect triggering of the morning and evening EMAs.

To address the limitations identified by Talbot [12] a revised protocol using both EMA and CPM (via Empatica^®^ E4 wristbands) was developed to explore the relationship between day-to-day occupational stress, musculoskeletal pain, and mental health in university staff in Australia [15]. To the authors’ knowledge, these day-to-day relationships have not been previously investigated. This paper reports a pilot study to test participant feasibility and acceptability of this revised protocol.

### Recruitment and procedures

University staff from a large metropolitan university in Melbourne, Australia were invited to participate in the study via email. An invite was also posted on Yammer (University internal social networking tool). Participants needed to be (1) predominantly engaged in sedentary work (2) at least 18 years of age (3) have access to a smartphone and a computer (4) work at least 80% of the full-time work week at the university (5) have permission from their supervisor to participate in the study. Ethics approval was granted by the La Trobe University Human Research Ethics Committee (HEC22236). No conflicts of interest were anticipated.

Participation involved three phases:

#### Preparatory phase

Participants completed a 15-min baseline questionnaire collecting information on demographic and employment characteristics, usual work hours, work environment risk factors, musculoskeletal pain, and mental health. Consent to participate was collected at the beginning of the questionnaire. A research staff member met with the participant to deliver the wristband and provide instructions on how to use it, including how to upload data and charge the device. They also provided instructions on the study protocol (e.g., duration, timing of questionnaires).

#### Main data collection phase

Participants were asked to wear the wristband during work hours for 10 work days and upload data daily. Each day participants were asked to complete a morning EMA (one hour after starting work with two reminders spaced 30 min apart); a daytime EMA (four hours after starting work with two reminders spaced 30 min apart); and an evening EMA (one hour after finishing work plus two reminders spaced one hour apart). To address the problems with the high frequency of surveys reported by Talbot [12], we reduced the number of surveys to three per day. Each EMA took 3–5 min to complete, with 21, 23 and 38 questions in the morning, daytime and evening surveys respectively. Most items were multiple choice format. Items asked about work environment risk factors (e.g., work demands, pace, autonomy), stress and musculoskeletal pain (specifically neck and back pain). Further detail about the measures can be found in the study protocol [15]. The timing of EMA questionnaires were tailored to participants’ usual work start and finish times and sent via SMS or email depending on participant preference. Time limits to complete each survey were applied to avoid overlap with subsequent surveys. The research team checked in with participants on day 2 to discuss any issues and again midway through data collection (day 5–6). Adherence was monitored and additional check ins with participants occurred as required to address difficulties with participation.

#### Concluding phase

A Researcher met with the participant to collect the wristband and conduct a brief semi-structured interview. Participants were asked about their experiences wearing the wristband and uploading the data and with completing the baseline and daily EMAs.

### Data analysis

Quantitative data were analysed in Stata/SE 18.0. Descriptive statistics were used to assess adherence to the study protocol (i.e., number of EMAs completed, days of wristband data uploaded). Linear regressions were used to assess whether there were any differences in EMA adherence (total, morning, daytime, evening) by gender (1 = man, 2 = woman), job type (1 = academic, 2 = professional staff) and caring responsibilities (e.g., for one or more dependents including child, adult with disability, ageing parent; 1 = yes, 0 = no). Qualitative data were analysed deductively using NVivo software (1.7.1), coding responses to the interview questions.

## Results

### Sample

Twenty-four participants were recruited. One withdrew before participation, leaving a final sample of 23. One participant withdrew on day 5, citing the study protocol as too onerous. Participants comprised 19 women and four men, 13 were academics and 10 were professional staff. Twelve reported having caring responsibilities.

### Wristband adherence

The wristband was worn for a mean of 9.7 days. There was a wide range from 2 to 12 days; however, removing the participant who withdrew on day 5 resulted in a smaller range from 7 to 12 days. Data were collected for approximately 7 h and 50 min per day (range: 4 h and 11 min to 10 h and 37 min). The mean number of “sessions” of data collection (indicating the number of times the wristband was switched on) was 11 (range: 2–25).

### EMA adherence

**Fig. 1:**
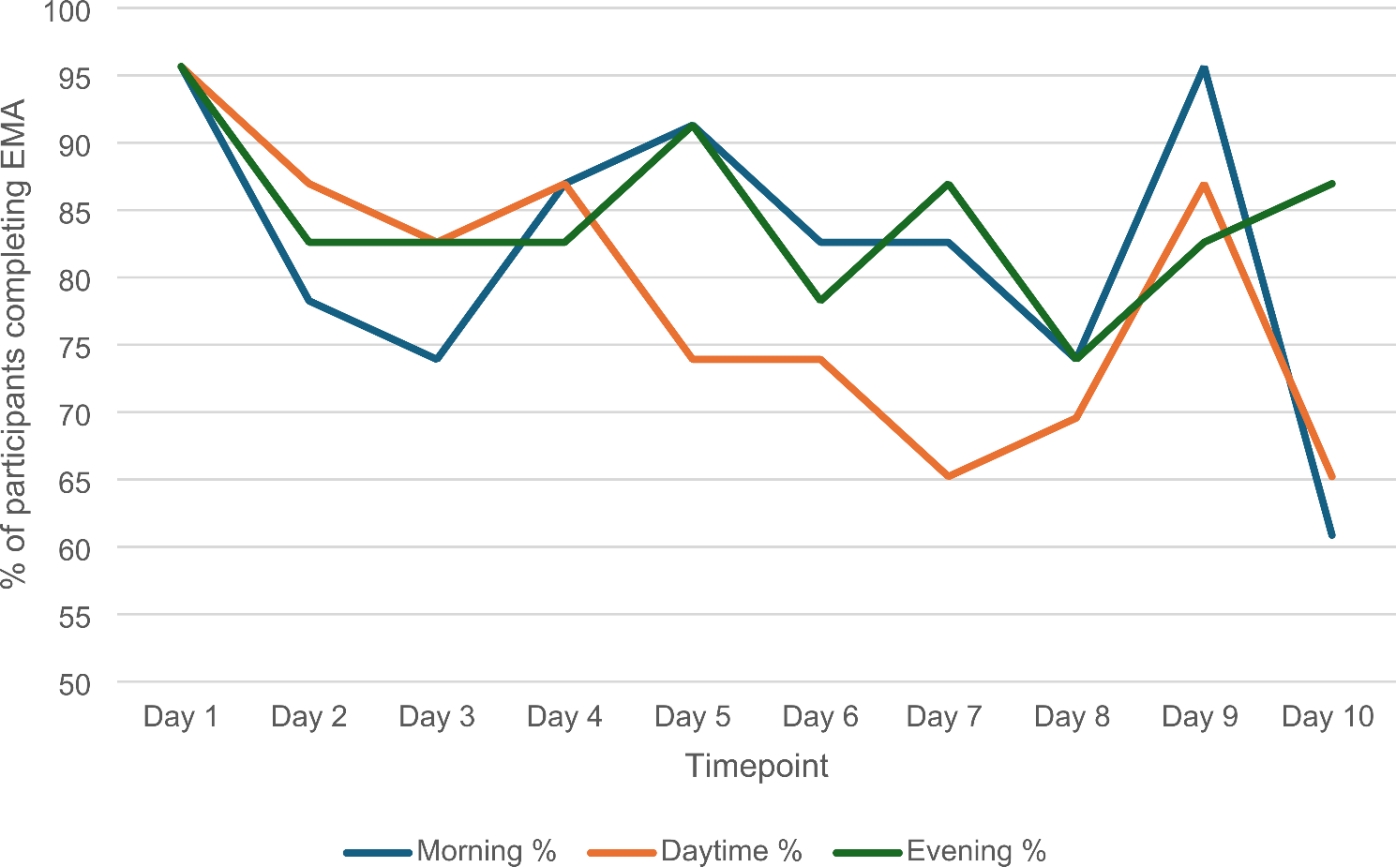
Number of participants completing EMAs across the 10-day study period

The mean number of EMAs completed across the study was 24.5, with one quarter (26%) completing all 30 surveys and almost three quarters (74%) completing 80% of surveys. The mean number of morning EMAs completed was 8.2 (out of 10), with the majority (87%) completing at least seven surveys (i.e., missing ≤ 3 surveys). The mean number of daytime surveys completed was 7.9, with 78% completing at least seven surveys. The mean number of evening surveys completed was 8.4, with 78% completing at least seven surveys. To assess whether there was any indication of a change in adherence over time a line graph was generated (Fig. 1). We observed initial enthusiasm with high completion rates for all 3 EMAs on day 1, followed by a drop on day 2. A slight downward trend is observed for the remainder of the study, with perhaps a stronger decline for the daytime survey. Participants with caring responsibilities completed fewer daytime EMAs than those without caring responsibilities (*β* = -2.79, CI -5.29, − 0.30, *p* = .03). No other differences in EMA adherence by participant characteristics were found.

### Participant experiences with study

Themes emerging from the interviews were associated with the prompts used: wristband perceptions and use, EMA timing and frequency, and reflections on participation. Many participants reported the wristband was easy to wear (n = 14) and data uploads were straightforward (n = 10). However, 10 reported that the wristband was obtrusive using words such as “clunky”, “uncomfortable” and “embarrassing”. Five participants commented it was inconvenient to wear their watch on the other hand or not at all …

“I tried putting my watch on the other arm but it just really annoyed me so I didn’t wear my watch and that was a little bit inconvenient sometimes” (Woman, Academic).

Participants reported using reminders on their phone or calendar to remember to wear the wristband or leaving it in a place where they would be unlikely to forget it (e.g., with work computer, work bag). Most participants (*n* = 12) uploaded the data every day or almost every day. Eight participants reported experiencing problems when uploading the data, such as receiving error messages that caused confusion. When this happened, participants usually contacted the research team for help and reassurance.

While most participants (n = 15) reported that the timing and frequency of EMAs were appropriate, some felt that the morning or daytime EMA was not necessary as change was minimal with respect to their work between the two timepoints. Related to this, some participants reported that it was difficult to answer certain items (e.g., those related to learning and using new skills) after being at work for an hour or when not much had changed in the short time between EMAs. Others found that the timing of EMAs did not always suit their work pattern with staff in university settings often working flexible hours (e.g., changing start and finish times, returning to work at night), making some items difficult to answer as they had not yet started or completed their day’s work. Completing particular EMAs (i.e., morning, daytime, or evening) was sometimes not possible due to competing work or non-work demands; however, many noted that the reminders were helpful, as they would have otherwise forgotten to complete the EMA.

“The nature of my work is such that is not very often 9 − 5. . I’ll get up early in the morning and do things for maybe 45 minutes or an hour and then go shower and have breakfast or exercise or whatever, so I didn’t bother putting it on then” (Male, Academic).

Four participants stated that participation in the study provided an opportunity to reflect on their experience of work and stress throughout the day. In particular, some academic participants reported that stress was often self-imposed, sometimes unnecessarily.

## Discussion

The modified protocol overcame many of the barriers to feasibility and acceptability identified by Talbot [12]. The length of the study (10 working days) was appropriate. Adherence to wearing the wristband was good; however, difficulties with turning the wristband on (resulting in periods with no physiological data collection) and uploading the data were common, as were reports that the wristband could be uncomfortable to wear or obtrusive. Smart watches or fitness trackers have been used to assess physiological symptoms in other research [e.g., 16, 17–19] and might provide an acceptable option for a scale up of this study protocol. Advantages of such commercially available devices are their comparatively lower cost to purchase and familiarity of use for many potential participants; however, further research is needed to ensure such devices collect valid and reliable data in different situations, and consideration needs to be given to how the data will be extracted.

Adherence to the EMA protocol was good, with a slight downward trend observed across the study duration, with some participants reporting that they were beginning to find participation onerous towards the end of the study. Those with, as opposed to without, caring responsibilities completed fewer daytime EMAs. While participants did not specifically identify caring responsibilities as a factor, we suggest that time pressures, arising from a less flexible finish time, may have contributed to this finding.

Participant comments highlighted some potential problems. Firstly, the flexible nature of work in a university means work hours are not always consistent across the week and people often work outside of their formalised work hours. Therefore, EMA timings based on usual work hours were not always suitable. Secondly, whilst three EMAs per day was considered acceptable, the timing of the morning EMA meant that work had just commenced, making some questions, such as having enough time to do the work well, irrelevant. Similarly, not much may have changed with respect to work in the relatively short time (three hours) between the morning and daytime EMAs, again making some questions difficult to answer. This was particularly true where change across the day may not be expected, such as learning and utilising new skills which often happens over a number of days or weeks in a university context and therefore may not be appropriate to be asked more than once per day. A further challenge with occupational stress is asking participants who may feel time pressured to take time to complete an EMA, which may increase their perceived stress level.

Difficulty answering some questions due to an individual’s job role was also noted; for example, academics often do not expect to receive feedback on their work throughout the day from their supervisor and the nature of their work means that workload pressures frequently result in needing to work longer, rather than faster. Lukan et al. [11] also noted difficulties caused by particular EMA questions and the present study corroborates the need for detailed consideration of EMA timing and questions tailored to the population and context.

## Conclusion

This study demonstrates that the revised study protocol was feasible and acceptable for the university workers in the Australian context. Further consideration needs to be given to the device used to collect physiological data. Additionally, modifications to EMA scales and items may be required to ensure they are relevant for the population of interest. This is especially important as EMAs are often very brief with minimal items and scales that are asked repeatedly across the study period. EMA frequency and timing should also be reviewed to ensure EMA delivery is appropriate to workers’ schedules and work tasks, which may require variation to be accommodated within the protocol.

### Limitations

Data were collected from university staff in one institution where staff work in predominantly sedentary roles. This may limit generalisability of results to those in other industry sectors or roles. As this was a pilot study, useable data were collected from 23 participants, again potentially limiting generalisability.

## Data Availability

Data available upon reasonable request from the authors.

## Abbreviations

CPA: Continuous Physiological Monitoring
EMA: Ecological Momentary Assessment
